# Should hospital managers read the orthopedic literature before surgeons? The example of femur fracture management

**DOI:** 10.1007/s10195-016-0427-6

**Published:** 2016-08-18

**Authors:** Alessandro Aprato, Denis Longo, Matteo Giachino, Gabriele Agati, Alessandro Massè

**Affiliations:** 1 San Luigi Hospital of Orbassano, University of Turin, Regione Gonzole 10, Orbassano, Turin, Italy; 2 University of Turin, C.so Massimo d’Azeglio 60, Turin, Italy

**Keywords:** Hip fractures, Elderly, Early treatment, Public healthcare

## Abstract

**Background:**

Early surgical intervention in the treatment of proximal femur fractures has been shown to significantly reduce mortality and complications. Our study intends to evaluate early surgery rates in a single-center analysis before the clinical advantages of early surgical intervention were demonstrated in the literature (G1), after the orthopedic team aimed to treat those fractures within 48 h (G2), and after early intervention became a primary objective for hospital management (G3).

**Materials and methods:**

The hospital charts of 894 proximal femur fractures in patients aged >65 years between 2008 and 2015 were analyzed in a single teaching hospital. The patients were allocated to three groups according to admission date, relative to the introduction of the different targets for early intervention. Our primary aim was to evaluate the differences in the rate of surgical treatment within 48 h in the three groups, and our secondary aim was to see if those differences influenced clinical outcomes.

**Results:**

The rate of treatment before 48 h was 23, 49 and 72 % in groups 1, 2 and 3, respectively (*p* < 0.001). There were no statistically significant differences between the three groups regarding time from surgery to discharge and perioperative mortality. The length of hospitalization was different only between groups 1 and 2.

**Conclusions:**

The adoption of an early treatment goal for proximal femur fractures by the orthopedic team significantly improved the results. However, it was only by introducing this goal into primary hospital management objectives that significantly increased the performance.

*Level of evidence* Level IV (retrospective case−control study).

## Introduction 

Hip fractures in the elderly represent the most common orthopedic injuries worldwide. In Italy, the incidence rate of hip fractures is approximately 1.4 fractures/1000 inhabitants per year, and ranges from 6.5−7.5/1000 individuals aged >65 years [1]. The number of people who will suffer this injury will largely increase over the following decades due to the aging population [1, 2]. Evidence suggests that surgery is the most effective treatment for femur fractures and recent guidelines report that early surgical treatment improves functional and clinical results [3–8]. Several published papers suggest a cut-off for surgery at 48 h and demonstrated that early surgical intervention reduces hospitalization and complications [5]. Most of the data were published over the past 10 years and the results and recommendations quickly spread throughout the world. Initially the orthopedic community started to discuss the implementation of practicing early surgical treatment of femoral neck fractures in 2007, supported by the first relevant literature. Since 2008, the Ministry of Health has introduced early surgical treatment for femur neck fractures as one of the indicators of hospital efficiency [9]. However, the orthopedic community originally struggled to achieve this goal without support from public health management [10]. In our region, the rate of early treatment for femur fractures was specifically included as one of the principal indicators of management performance at each single hospital in only 2012 [11].

The aim of this study is to compare the timing for femur treatment in a teaching hospital during three time periods—the first two time-frames are before and after the orthopedic team became involved in a training program focused on the efficacy of early treatment (respectively from 2008−2010 and from 2011−2013), while the third period (from 2013−2015) started when early treatment was included as an indicator of hospital management performance. The secondary aim is to evaluate if the changes influenced perioperative mortality and length of hospitalization.

## Materials and methods

### Settings

Between 2008 and 2015, 894 femur fractures in patients aged >65 years were treated in a single teaching hospital. The patients were allocated to three groups according to hospital admission date—the first group (G1) from 1 January 2008 to 31 December 2010, the second group (G2) from 1 January 2011 to 31 December 2012, and the third group (G3) from 1 January 2013 to 31 December 2015. The subdivision date of the first two groups corresponds to the enrollment of the orthopedic team on 1 January 2010 into the European Quality of Care Pathways Study on Proximal Femur Fracture (EQCP-PFF) [12]. This study was an international multicentric research project launched by the European Pathway Association (E-P-A) [13]. The overall project consists of training orthopedic teams, and focusing on the processes and outcomes of a care pathway for patients with proximal femur fractures (PFFs).

The third group includes patients from 1 January 2013, when our region included the rate of early treatment for femur fractures to the principal indicators of management performance at each single hospital [14].

### Data collection

Hospital charts were retrospectively reviewed after patients had given informed consent to the use of their data. Data on demographics, diagnoses (according to the AO classification), type of surgical treatment, American Society of Anesthesiologists (ASA) physical status classification, time from trauma to surgery, time from surgery to discharge, perioperative mortality and transfusion requirement were retrieved from medical records and recorded in a custom-made database [15, 16].

The present study was approved by the Institutional Review Board and was conducted in accordance with the ethical standards laid down in the 1964 Declaration of Helsinki and its later amendments.

All data were analyzed with standard descriptive statistics. Univariable analysis was performed to compare the three groups with regard to age, type of fracture, ASA score, treatment before/after 48 h, time from trauma to surgery, time from surgery to discharge, age at mortality and transfusion rate. Chi-squared test or Fisher’s exact test for categorical outcomes, and Student’s *t* test or Mann–Whitney test for continuous outcomes were used. The Kolmogorov–Smirnov test was used to determine whether data were normally distributed. *p* values <0.05 were considered statistically significant. All analyses were performed using Stata version 12 (Stata Corporation, College Station, Texas, USA).

## Results

In Table 1, the number of patients, mean age, rate of medial/lateral fracture and mean ASA score are compared. No significant differences were found between the three groups (all *p* values >0.05).

**Table 1:** Comparison of demographic data

	G1	G2	G3
No. of patients	324	223	337
Age (years)	87 (SD 8)	87 (SD 6)	85 (SD 7)
Number of medial fractures (rate)	203 (63 %)	151 (68 %)	214 (64 %)
Mean ASA score	2.4 (SD 0.9)	2.5 (SD 0.7)	2.7 (SD 0.7)

The rate of treatment before 48 h was 23, 49 and 72 % in G1, G2 and G3, respectively. Those differences were significant (*p* value <0.001; Pearson’s chi-squared test 154,144; degrees of freedom 2).

**Fig. 1:**
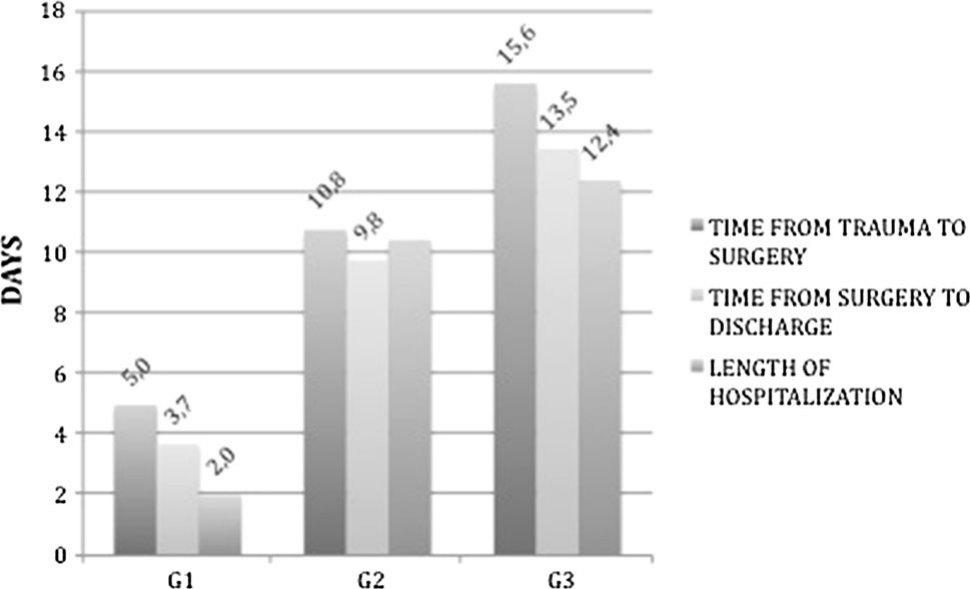
Mean time from trauma to surgery, mean time from surgery to discharge, and length of hospitalization for the three groups

**Fig. 2:**
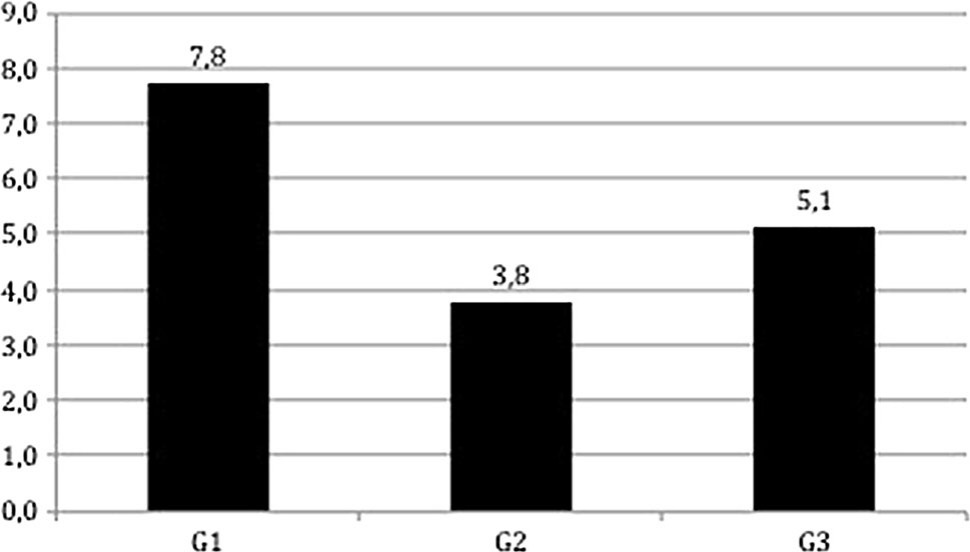
Mortality according to group

The mean time from trauma to surgery, mean time from surgery to discharge and length of hospitalization for the three groups are shown in Fig. 1. Differences of time from trauma to surgery were statistically significant between G1 and G2 (*p* < 0.001), and between G2 and G3 (*p* = 0.01). No difference was statistically significant (all *p* values >0.05) between the three groups regarding time from surgery to discharge. The length of hospitalization was significantly reduced between G1 and G2 (*p* = 0.002) but not between G2 and G3 (*p* = 0.126). Overall time from trauma to surgery was 3.51 days (SD 4.10), time from surgery to discharge was 10.39 days (SD 6.44) while length of hospitalization was 13.86 days (SD 7.93). Overall perioperative mortality was 5.8 % (48/831). The mortality rate for the three groups is shown in Fig. 2.

## Discussion

Early treatment of femoral fractures has become the standard of care over the past 5 years. In Italy, this goal has been difficult to achieve and in 2012 the national rate of early treatment was 40.5 % [10]. In order to help the surgeons to achieve this goal, early treatment of femur fractures was introduced into the main objectives of hospital general managers in 2013.

Our study aimed to evaluate the performance before the evidence supporting the early treatment of femur fractures influenced the surgeons, after this process, and eventually after the introduction of this criterion into the evaluation of management performance at each single hospital.

According to our results, the training process for surgeons showed a significant improvement in the rate of early treatment (from 23−49 %). However, the introduction into hospital management evaluation criteria was even more effective, significantly raising the rate to 72 %. Therefore, the impact of this last decision was crucial to achieve the standard of care suggested in the literature.

We think the reasons behind the results are largely due to the implementation of dedicated procedures for surgery in PFF patients. At the time when only surgeons were influenced by the findings in the literature, the anesthesiologists evaluated the patient in the Accident and Emergency (A&E) Department and the surgery was performed either in the emergency operating room on the same day if free or in the first free slot in the orthopedic operating room. In our hospital, this procedure alone leads to a suboptimal early treatment rate (G2) underlining the difficulties of orthopedic surgeons alone in performing early surgery without the involvement of anesthesiologists, bed managers and emergency doctors.

The co-operation between those parties was possible only when guided, promoted and supervised actively by the hospital management. In our department after 2013 the hospital management decided to promote the early treatment for femur fractures in three ways. First, the rate of early femoral fracture fixation was included into the performance evaluation of anesthesiologists. Second, patients requiring surgery before 48 h received priority for hospitalization in case of overbooking in the A&E department. Third, the protocol for emergency criterion assignment was revised by changing from class B (before 48 h) to class A (before 24 h) in the emergency operating room on the subsequent day if the femur fracture had not been treated on the first day from arrival in A&E. No economic resources have been used to improve the early treatment rate by the hospital management.

The length of hospitalization did not show significant differences during the three periods, which reflects the national difficulties in patient discharge from surgical units. Although the time from trauma to surgery was significantly reduced over time, no improvement was achieved in the reduction of the postoperative period. Therefore, although the overall economic burden of the fractures may be reduced by the reducing the complications, the direct costs of hospitalization have not changed.

Our results show that perioperative mortality was not significantly influenced by the increase in early treatment rates. This may reflect one of the major limitations of this study, i.e., early treatment is proved to influence mainly medium and long-term mortality, but not perioperative findings [7].

The main limitation of this study is the retrospective design. Furthermore, record analysis was performed in a single hospital reviewing only clinical charts.

Organizational and technical factors are more difficult to extract from patient records and data on hospitalization length are largely influenced by non-medical issues; these are factors which may limit the strength of our conclusions. Mortality after discharge has not been evaluated and most of the published studies focused on those data.

In conclusion, we think a direct connection between government health departments and the orthopedic community should be established to improve the transmission of literature to clinical practice, invariably through cooperation with local administrations.

As shown by our results, only this co-operation may transform recommendations in the literature into clinical standards of care.
